# Efficient Spatiotemporal Attention Network for Remote Heart Rate Variability Analysis

**DOI:** 10.3390/s22031010

**Published:** 2022-01-28

**Authors:** Hailan Kuang, Fanbing Lv, Xiaolin Ma, Xinhua Liu

**Affiliations:** Hubei Key Laboratory of Broadband Wireless Communication and Sensor Networks, School of Information Engineering, Wuhan University of Technology, Wuhan 430070, China; kuanghailan@whut.edu.cn (H.K.); lvfanbing@whut.edu.cn (F.L.); liuxinhua@whut.edu.cn (X.L.)

**Keywords:** remote photoplethysmography, heart rate variability, 3D convolutional neural network (3DCNN), depth-wise separable convolution, attention mechanism

## Abstract

Studies have shown that ordinary color cameras can detect the subtle color changes of the skin caused by the heartbeat cycle. Therefore, cameras can be used to remotely monitor the pulse in a non-contact manner. The technology for non-contact physiological measurement in this way is called remote photoplethysmography (rPPG). Heart rate variability (HRV) analysis, as a very important physiological feature, requires us to be able to accurately recover the peak time locations of the rPPG signal. This paper proposes an efficient spatiotemporal attention network (ESA-rPPGNet) to recover high-quality rPPG signal for heart rate variability analysis. First, 3D depth-wise separable convolution and a structure based on mobilenet v3 are used to greatly reduce the time complexity of the network. Next, a lightweight attention block called 3D shuffle attention (3D-SA), which integrates spatial attention and channel attention, is designed to enable the network to effectively capture inter-channel dependencies and pixel-level dependencies. Moreover, ConvGRU is introduced to further improve the network’s ability to learn long-term spatiotemporal feature information. Compared with existing methods, the experimental results show that the method proposed in this paper has better performance and robustness on the remote HRV analysis.

## 1. Introduction

Abundant research has shown that autonomic nerve activity is associated with many diseases, and some cardiovascular and cerebrovascular diseases are more closely related to autonomic nerve activity. Dekker et al. also proved that autonomic nerve activity can effectively regulate heart rate changes [1]. Through HRV analysis, key metrics of autonomic nervous activity can be obtained, which can be further used to predict or indicate emotional state, cardiovascular disease, sleep quality, etc. Therefore, HRV analysis has great practical significance.

**Figure 1:**
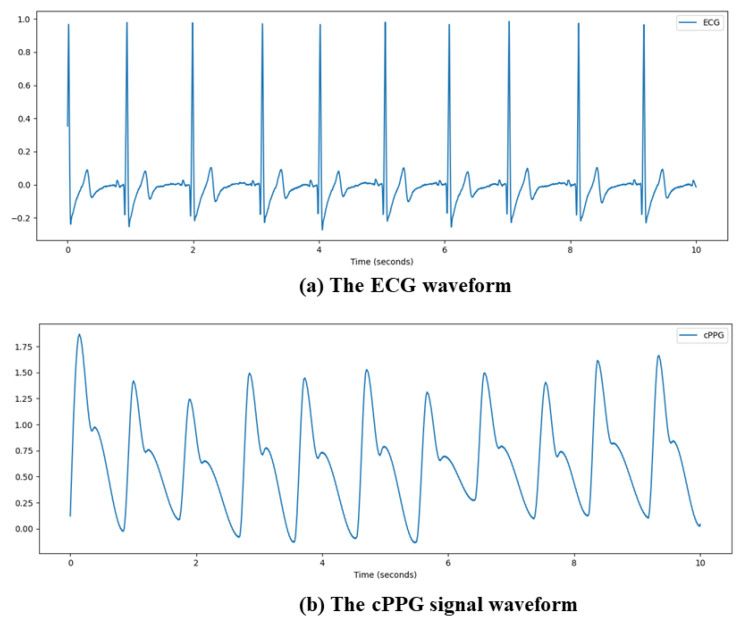
Signal waveform obtained by ECG and cPPG technology.

In clinical practice, the two most commonly used HRV analysis methods are electrocardiography (ECG) and contact photoplethysmography (cPPG). ECG is a technique that uses an electrocardiograph to record the changes of electrical activity generated by each cardiac cycle from the body surface. The heart is excited by the pacemaker, atrium, and ventricle, successively, in each cardiac cycle. Along with the changes in the electrocardiogram bioelectricity, various forms of potential changes are drawn from the body surface through the electrocardiograph. ECG is an objective indicator of the occurrence, propagation and recovery of cardiac excitation. cPPG refers to the use of an optical heart rate sensor to emit light of a specific color and wavelength through the luminous LED on the sensor, which is incident into the epidermal skin of specific parts of the human body, and then receive the corresponding reflected light or incident light according to the photosensitive sensor to achieve the purpose of detecting the pulse signal. The signal waveforms obtained by ECG and cPPG technology are shown in Figure 1. Since both ECG and cPPG need to be in contact with the skin surface, the use of these two methods for HRV analysis may be limited. Contact may not always be suitable for applications such as driver pressure detection, behavior sensing, and monitoring for symptoms of sudden cardiac death in neonatal care units, so the use of ECG and cPPG for HRV analysis may be limited. If non-contact HRV analysis is proven to be valid, many applications will be able to benefit.

The principle behind the non-contact physiological measurement based on rPPG is that the amount of light absorbed by the skin changes periodically with the periodic changes in blood volume caused by the heartbeat. Its external feature is the periodic change of the skin color caused by the heartbeat, and this change can be captured by the camera. By analyzing the color changes of the human facial skin in the video, the rPPG signal can be obtained, and then, the physiological measurement such as HRV can be obtained [2,3,4].

At present, the research on non-contact physiological measurement is still in its infancy. In the early days of rPPG methods, principal component analysis (PCA) or independent component analysis (ICA) were usually used to decompose the blood volume pulse (BVP) signal [5,6,7,8] or CHROM [9] and POS [10,11] et al. were used to extract BVP signal through color space changes to estimate heart rate (HR). In these studies, most methods can be regarded as two-stage: firstly, the face must be detected or tracked to obtain the region of interest (ROI) area, then, the color change signal of the skin in the ROI area must be obtained, and finally, the frequency must be analyzed in order to estimate the corresponding average heart rate. However, these methods have two shortcomings that we need to pay attention to. First, their research is based on pure prior knowledge to customize which parts of the face area are the most effective, but the prior knowledge is not necessarily valid in different datasets. Second, these methods are designed to manually process features or design filters, which may not be well generalized.

With the continuous maturity and development of deep learning in the field of image processing, researchers have also begun to use deep learning methods for non-contact heart rate (HR) measurement [12,13,14,15]. DeepPhys [12] is the first end-to-end method for estimating heart rate and respiratory frequency from video and verifies the feasibility of recovering rPPG signals from frame value differences. At the same time, an attention mechanism is introduced to improve the accuracy of rPPG signal extraction. HR-CNN [13] proposed a two-stage HR prediction model, which consists of two parts: an extractor and a predictor. The extractor extracts the rPPG signal and passes it to the predictor to predict HR. RhythmNet [14] uses CNN and gated recurrent units to form a spatiotemporal representation for estimating HR. Meta-rppg [15] uses unlabeled samples for self-monitoring weights to resolve unforeseen distribution changes during deployment, thereby improving the accuracy of HR estimation during deployment. Most of these methods only predict relatively simple physiological parameters such as HR. To analyze more complex physiological measurement such as HRV, we need to be able to obtain high-quality rPPG signals. In order to obtain high-quality rPPG signals, it is important to learn the spatiotemporal features from video data.

**Figure 2:**
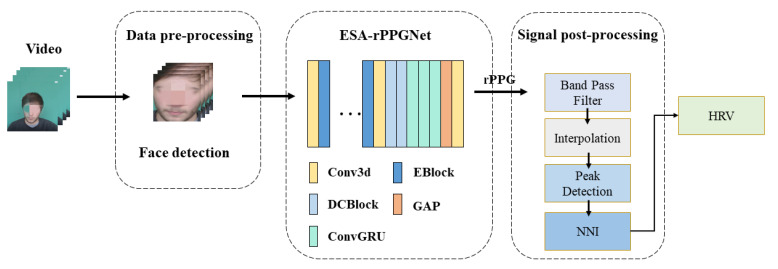
Proposed HRV analysis framework using ESA-rPPGNet.

In order to solve the above problems, this paper conducts spatiotemporal modeling of facial video, and an efficient spatiotemporal attention network ESA-rPPGNet is designed to recover high-quality rPPG signals to accurately locate the peak of each heartbeat, which improves the accuracy of HRV analysis and reduces the time complexity of the network. Figure 2 shows the framework of the proposed rPPG signal measurement method and steps for analysis. See Chapter 3 of the article for details.

The main contributions of this paper are as follows: (1) An end-to-end method for recovering rPPG signals from facial videos is proposed. This method not only effectively improves the quality of the recovered rPPG signal, but also greatly reduces the time complexity of the network. (2) A plug-and-play lightweight attention block, 3D Shuffle Attention (3D-SA), is proposed which improves the network’s ability to capture pixel-level dependencies and inter-channel dependencies. 3D-SA allows the network to focus on useful features, suppress unimportant features, and improve the accuracy of the network. (3) Tests on different datasets prove that this method can recover rPPG signals with accurate peak time locations that match with ground truth signals and is superior to existing methods for remote HRV analysis.

## 2. Related Work

Recently, most research on non-contact physiological measurement has focused on the average HR. The average HR measurement is mainly used to calculate the total number of heartbeats in a given time period. Compared with HRV analysis, average HR measurement is a very simple way to describe heart activity. If we want to be able to describe heart activity more precisely, we need to perform HRV analysis.

**Figure 3:**
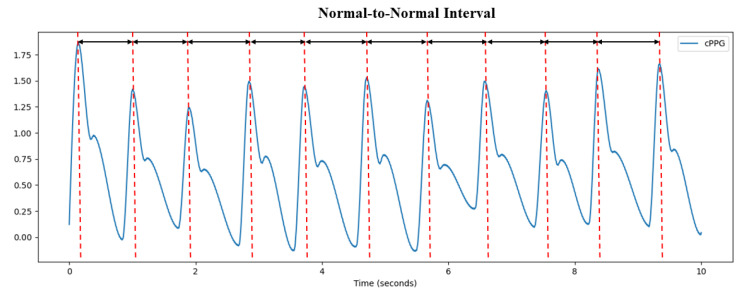
Normal-to-normal interval (NNI) obtained from rPPG signal.

There are three steps in non-contact HRV analysis. First, the rPPG signal must be obtained from the facial video. Then, the peak time locations of the rPPG signal is obtained by the peak detection algorithm. By measuring the time interval between the peaks, these time intervals form a set of sequences, as shown in Figure 3. Finally, HRV analysis is performed through the time interval sequence.

Since HRV is calculated based on the change of the heartbeat interval, it is very important to accurately detect and locate each peak of the rPPG signal. This makes some methods that achieve good results in HR prediction tasks not effective in HRV analysis. In past few years, some methods have tried non-contact HRV analysis from video. Rodriguez et al. [16] first detected faces in videos and obtained the ROI, then processed and analyzed the signal of the green channel to obtain the rPPG signal; finally, the rPPG signal was filtered through a band-pass filter to obtain a clean rPPG signal. The authors of this study used their own private dataset for HRV metrics evaluation. Finžgar and Podržaj in [17] proposed a method to recover rPPG signal through wavelet transform and custom beat interval filtering. They proved good correlation between time domain ultra-short term HRV measurements from rPPG and PPG through a series of experiments and evaluated it on the public dataset PURE [18]. Li et al. proposed a method to improve the accuracy of heartbeat interval detection by using the slope sum function in [19], and evaluated the HRV features that can be used for emotion detection in the public dataset UBFC-rPPG [20]. The results proved the effectiveness of their method. Amogh Gudi et al. proposed a refined and efficient real-time rPPG extraction method in [21]. This method has novel filtering and motion suppression functions. First, they use the images captured by the webcam camera to detect and model the face and extract a single BVP signal from the skin pixels of ROI by using the POS method; at the same time, they track the head movement to suppress the noise generated by motion. The high-quality rPPG signal obtained by their method can not only estimate the heart rate, but can also analyze the heart rate variability.

Most of the above methods use color space transformation or blind source separation to extract pulse signals from RGB video for physiological measurement. Although these methods can effectively improve the accuracy of physiological measurements, they all require some prior assumptions, and these assumptions may be invalid in some cases, such as changes in illumination and head movement.

Recently, in order to overcome the limitations of traditional models, researchers have begun to try to use deep learning for HRV analysis. Song et al. [22] proposed a generative adversarial network model, PulseGAN, which can effectively improve the quality of the rPPG signal. In the paper, the rPPG signal is extracted from the RGB video by CHROM, and then the rPPG signal is enhanced by the PulseGAN model. Finally, the temporal metrics of HRV are evaluated on the UBFC-rPPG dataset. Xuesong Niu et al. proposed a cross-validation feature separation strategy in [23]. First, the physiological features and non-physiological features are separated, and then the extracted physiological features are used for multi-tasking physiological measurement, and comprehensive experiments are carried out on different large-scale datasets. The results verify the robustness of the method. Shu Liu’s work, [24], proposed a tree-based probabilistic fusion neural network. This method first creates probability embedding from the data, and then uses the neural network for further fusion and processing. Shu Liu’s work is the first to use facial videos based on driving data collected from public roads in real driving scenarios for HRV outlier detection.

Now, many methods use 2DCNN for feature extraction. Although 2DCNN has very good performance in the task of image classification and detection, such a model discards the time features, which provides very important information in the task of video processing. Moreover, the rPPG signal is essentially a time series, and temporal context is crucial for rPPG signal extraction.

**Figure 4:**
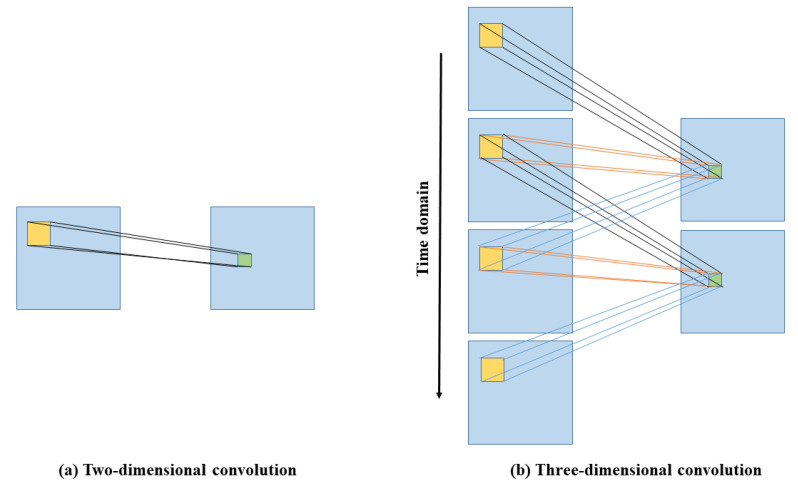
Comparison of 2D (**a**) and 3D (**b**) convolutions. In 3D convolution, the same 3D kernel is applied to overlapping 3D cubes in the input video to extract motion features.

Due to the excellent spatiotemporal feature extraction capability of spatiotemporal networks, they play a vital role in many video-based tasks. 3DCNN, as a spatiotemporal network, has been widely used in video processing tasks because of its better video spatiotemporal feature extraction capability than 2DCNN [24], as shown in Figure 4. Yu et al. first proposed a network constructed by 3DCNN in 2019, PhysNet [25], which can recover accurate rPPG signals from original facial videos and evaluated the measurement metrics of LF, HF and LF/HF of HRV. In Yu’s method, the C3D network architecture is used for construction. The disadvantage of this network architecture is that the time complexity is very high, which makes it difficult for this kind of network to be applied to resource-constrained devices.

## 3. Materials and Methods

### 3.1. Data Pre-Processing

**Figure 5:**
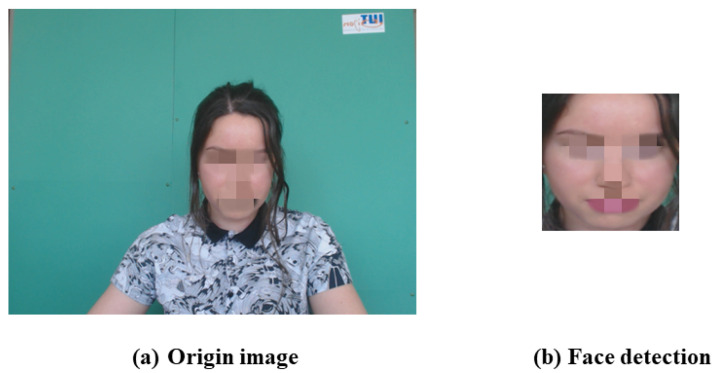
Face detection using MTCNN algorithm.

To reduce the noise caused by the irrelevant background and the motion artifacts in the videos, we use the face as the ROI for subsequent calculation. The face detection algorithm MTCNN [26] is used to detect the face of the videos in the dataset, as shown in Figure 5. In order to improve the processing speed of face detection, after recognizing the face position coordinates of the first frame, we store them. The same face position coordinates are used for the next 10 frames, and then the algorithm performs face detection again and updates the face position coordinates, and so on.

Since the ground truth PPG signals also have some noise, to alleviate the difficulty of training, the ground truth PPG signals are processed with a band-pass finite impulse response (FIR) filter with a cutoff frequency range of 0.5–4 Hz, which covers the human heart rate range 0.5–2.5 Hz. It not only retains the second harmonic and signal information in the original signal but also removes noise and smoothes the curve. To improve the training efficiency, we further normalize the filtered signals. Because the videos in the dataset used in this paper are all 30 FPS, in order to ensure the one-to-one correspondence between the ground truth PPG and the videos, we also downsample the PPG signal to 30 Hz.

### 3.2. 3D Depth-Wise Separable Convolution

**Figure 6:**
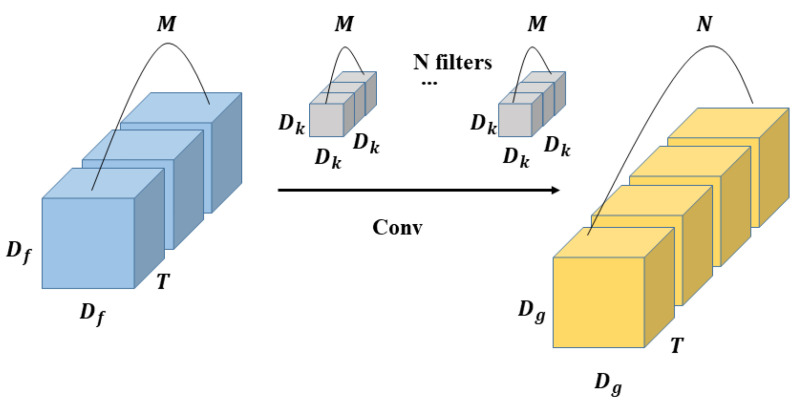
The process of standard 3D convolution.

The calculation process of the standard 3D convolution is that the convolution kernels extract features from the input feature maps, and then combine the extracted features into new feature maps for output. As shown in Figure 6, the size of input feature maps is $D_{f}\times D_{f}\times T\times M$, where $D_{f}$ is the length and width of the input feature maps, T is the temporal dimension, and M is the number of input channels. The size of output feature maps is $D_{g}\times D_{g}\times T\times N$, where $D_{g}$ is the length and width of the output feature maps, T is the temporal dimension, and N is the number of output channels. The size of the filters is $D_{k}\times D_{k}\times D_{k}\times M$, which is composed of M convolution kernels. The length, width and temporal dimension of the convolution kernels are all $D_{k}$. In the standard 3D convolution, the number of filters are the same as output channels, and the number of convolution kernels that compose the filter are the same as input channels.

Then the standard 3D convolution multiplication calculation amount is as follows:

(1) $$D_{k}\times D_{k}\times D_{k}\times D_{g}\times D_{g}\times M\times N\times T$$

**Figure 7:**
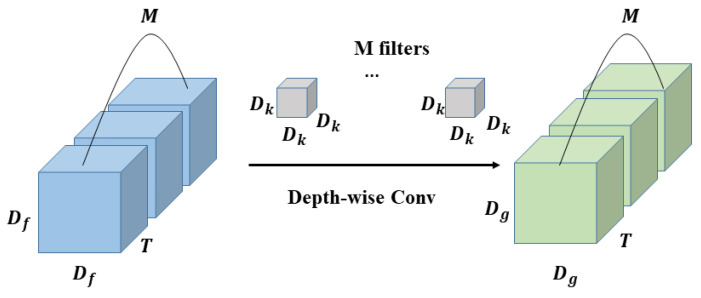
The process of 3D depth-wise convolution.

**Figure 8:**
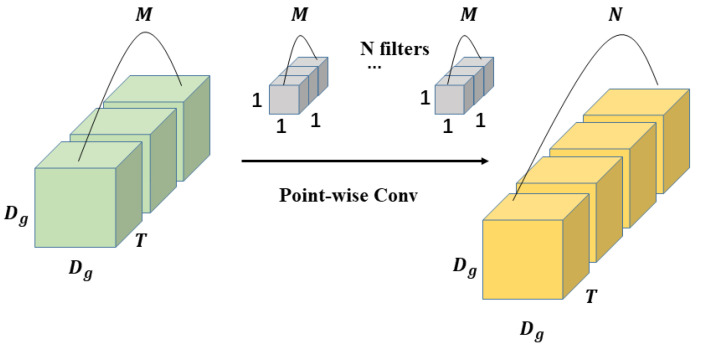
The process of 3D point-wise convolution.

The main difference between 3D depth-wise separable convolution and 2D depth-wise separable convolution is that 3D depth-wise separable convolution has one more temporal dimension. 3D depth-wise separable convolution decomposes the standard 3D convolution into two stages, depth-wise convolution (DWC) and point-wise Convolution (PWC), as shown in Figure 7 and Figure 8.

In depth-wise convolution, each channel of the input feature maps will be treated as a separate feature map, and each feature map uses the filter composed of only one convolution kernel to perform a convolution operation with it to obtain the same number of output feature maps as the input. The number of filters are the same as output channels. The final output can be obtained by superimposing these feature maps, and the calculation is as follows:

(2) $$D_{k}\times D_{k}\times D_{k}\times D_{g}\times D_{g}\times M\times T$$

In the above process, since the number of input channels is the same as the output, the feature map cannot be expanded. Moreover, this operation independently performs convolution operations on each channel of the input layer, and it does not effectively use the feature information of different channels in the same spatiotemporal position. In order to solve these problems, it is necessary to use the following point-wise convolution to combine these features.

In point-wise convolution, filters composed of multiple 1×1×1 convolution kernels are used to perform further feature extraction on the feature maps generated by depth-wise convolution. It is very similar to the standard 3D convolution process, except that the size of the convolution kernels that compose the filters used for point-wise convolution is all 1×1×1. After point-wise convolution, the feature maps generated in the previous step will be weighted and fused in the depth direction to obtain the final output feature maps of the depth-wise separable convolution. The calculation of 3D point-wise convolution is as follows:

(3) $$1\times 1\times 1\times D_{g}\times D_{g}\times M\times N\times T$$

Add the calculation amount of depth-wise convolution and point-wise convolution together to get the calculation of 3D depth-wise separable convolution:

(4) $$D_{k}\times D_{k}\times D_{k}\times D_{g}\times D_{g}\times M\times T+D_{g}\times D_{g}\times M\times N\times T$$

The ratio of the calculation amount of 3D depth-wise separable convolution to the calculation amount of standard 3D convolution is:

(5) $$\frac{\left(4\right)}{\left(1\right)}=\frac{1}{N}+\frac{1}{D_{k}^{3}}$$

From the above calculation, it can be seen that the calculation amount of 3D convolution is mainly concentrated in the process of feature extraction. Here, 3D depth-wise separable convolution reduces the number of feature extractions and increases the number of features merging by decomposing the convolution into depth-wise convolution and point-wise convolution, which greatly reduces the calculation of the model while having a small impact on the accuracy of the network. It is precisely because of this advantage of the depth-wise separable convolution that it is used as the basic convolution module in ESA-rPPGNet.

### 3.3. 3D Shuffle Attention Block

Attention mechanism first appeared in the fields of natural language processing and machine translation, and achieved good results. Through some scholars’ explorations, the attention mechanism has gradually been applied in the field of computer vision. The attention mechanism in deep learning is similar to the attention mechanism of human vision; that is, it focuses on important points in a lot of information, selects key information, and ignores other unimportant information. Adding the attention mechanism to the convolutional neural network can effectively improve the performance of the network.

The attention mechanism in computer vision is mainly divided into two categories, spatial attention and channel attention, which are used to capture pixel-level relationships and channel-level relationships. For 2DCNN, there are already many outstanding attention modules, such as SE block, based on channel attention [27] and CBAM [28], that combines channel attention and spatial attention. However, they all have their own shortcomings. SE block only uses channel attention, and does not make full use of the correlation between spatial attention and channel attention. Although CBAM combines channel attention and spatial attention, it inevitably increases the calculation and size of the model.

**Figure 9:**
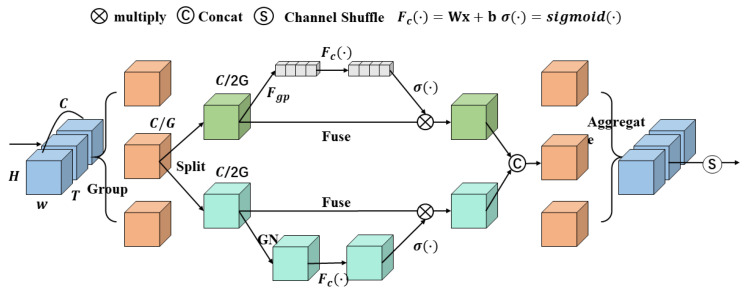
An overview of the proposed SA module. The C channels are divided into G groups and each group has C/G channels. “Split” means “channel split”. It adopts “channel split” to process the sub-features of each group in parallel. Each group is divided into two sub-features and each sub-feature has C/2G channels. $F_{gp}$ means to use global average pooling (GAP) to generate channel-wise statistics; then, $F_{c}$ uses a pair of parameters to scale and shift the channel vector. σ means to use the sigmoid activation function to create a compact feature to guide more precise and adaptive channel selection. Group norm (GN) is adopted to generate spatial-wise statistics. “Fuse” means keep the tensor unchanged, directly as input. The two branches are then concatenated. After that, all sub-features are aggregated, and finally, SA utilizes a “channel shuffle” operator to enable information communication between different sub-features.

To solve these problems, shuffle attention (SA) [29] first obtains multiple sub-features by grouping channel dimensions. Then, SA uses the channel and spatial attention mechanism for each sub-feature at the same time. Finally, SA gathers all the sub-features together and uses the channel shuffle [30] operation to fuse the sub-features of different groups. Based on the SA module, this paper constructs a 3D shuffle attention (3D-SA) module for 3DCNN, as shown in Figure 9.

Consider the input feature map $X\in R^{C\times T\times H\times W}$, where *C*, *T*, *H*, *W* indicate the number of channels, temporal dimension, spatial height and width, respectively. First, the feature map *X* is divided into *G* groups along the channel dimension, i.e., $X=\left[X_{1},...,X_{2}\right],X_{k}\in R^{C/G\times T\times H\times W}$. Each sub-feature $X_{k}$ gradually captures specific semantics during the training process. For each $X_{k}$, we will divide it into two branches along the channel dimension, i.e., $X_{k1},X_{k2}\in R^{C/2G\times T\times H\times W}$. These two branches use channel attention and spatial attention, respectively, to generate channel attention maps and spatial attention maps. In this way, the model can know what to pay attention to and where it is meaningful to pay attention.

In order to design a more lightweight attention module in the channel attention branch, instead of directly using the SE block, a lightweight implementation is used [29]. In order to be able to embed the global information, global average pooling (GAP) is used to generate the channel-wise global statistical information as $s\in R^{C/2G\times 1\times 1\times 1}$, which reduces the temporal and spatial dimensions T×H×W of $X_{k1}$:

(6) $$s=F_{gp}\left(X_{k1}\right)=\frac{1}{T\times H\times W}\sum_{i=1}^{T}\sum_{j=1}^{H}\sum_{k=1}^{W}X_{k1}\left(i,j,k\right)$$

In addition, a simple gating mechanism is implemented by using the sigmoid activation function to create a compact feature to guide more precise and adaptive channel selection. Therefore, the final output of the channel attention branch can be obtained by

(7) $$X_{k1}^{\prime }=\sigma \left(F_{c}\left(s\right)\right)\cdot X_{k1}=\sigma \left(W_{1}s+b_{1}\right)\cdot X_{k1}$$

where $W_{1}\in R^{C/2G\times 1\times 1\times 1}$ and $b_{1}\in R^{C/2G\times 1\times 1\times 1}$ are parameters used to scale and shift s.

Unlike channel attention, which pays more attention to “what ”, spatial attention pays more attention to “where ”. The information obtained by spatial attention can be used as a supplement to the information obtained by channel attention. In the spatial attention branch, $X_{k2}$ is firstly group normalized (GroupNorm, GN) [31] to obtain spatial level information [29]; then $F_{c}\left(.\right)$ is used to enhance the spatial features, and finally, by using the sigmoid activation function, a simple gating mechanism is created. The final output of the spatial attention branch can be obtained by

(8) $$X_{k2}^{\prime }=\sigma \left(W_{2}\cdot GN\left(X_{k2}\right)+b_{2}\right)\cdot X_{k2}$$

where $W_{2}\in R^{C/2G\times 1\times 1\times 1}$ and $b_{2}\in R^{C/2G\times 1\times 1\times 1}$.

After getting the output of the channel attention and the spatial attention branch, the output of the two branches are concatenated along the channel dimension to get the same number of channels as the number of input, i.e., $X_{k}^{\prime }=\left[X_{k1}^{\prime },X_{k2}^{\prime }\right]\in R^{C/G\times T\times H\times W}$. When all the sub-features are aggregated, the channel shuffle operation similar to ShuffleNet v2 [30] is used to make the information between the cross-groups flow along the channel dimension, and finally the output of the 3D-SA module is obtained. Since the output dimension of the network is the same as the input dimension, the 3D-SA block can be easily applied to the 3DCNN network model.

In the non-contact rPPG extraction task, we need the network to be able to learn the skin color change caused by changes in the pulse on the skin of the face. By introducing an attention mechanism, our network can focus more on the skin color changes, ignoring the noise caused by eyes, mouths and other objects. As a result, the effectiveness and robustness of the network can be improved.

### 3.4. Recurrent Neural Network ConvGRU

A recurrent neural network (RNN) is a special neural network structure which is different from ordinary 2DCNN. It not only considers the input at the current moment but also gives the network the ability to remember the previous content. This means that RNN has a very good effect in dealing with time-series problems. However, RNN also has some serious shortcomings, such as gradient disappearance and gradient explosion. In order to solve these problems, a series of improved algorithms have appeared, of which there are two main ones: long short-term memory (LSTM) and gated recurrent unit (GRU).

As a variant of LSTM, GRU combines the forget gate and output gate into a single update gate. Compared with LSTM, it has similar performance but requires lower memory and is easier to train [32]. The GRU allows each cyclic unit to adaptively capture the dependencies of different time scales, and its calculation method is as follows:

(9) $$z_{t}=\sigma \left(W_{z}x_{t}+U_{z}h_{t-1}\right)$$

(10) $$r_{t}=\sigma \left(W_{r}x_{t}+U_{r}h_{t-1}\right)$$

(11) $${\tilde{h}}_{t}=\tanh\left(Wx_{t}+U\left(r_{t}⊙h_{t-1}\right)\right.$$

(12) $$h_{t}=\left(1-z_{t}\right)h_{t-1}+z_{t}{\tilde{h}}_{t}$$

where ⊙ is element-wise multiplication. $z_{t}$ is the update gate, which is used to control the degree to which the state information of the previous time is substituted into the current state. The larger the value of the update gate, the more the state information of the previous time is brought in. σ is the sigmoid activation function. $r_{t}$ is the reset gate, which is used to control the degree of ignoring the state information of the previous time. The more obvious its value is, the more it is ignored. ${\tilde{h}}_{t}$ is the candidate hidden layer, similar to ${\tilde{c}}_{t}$ of LSTM, which can be regarded as new information at the current time.

However, for a video task, the input of the convolutional feature map is a three-dimensional tensor, that is, spatial dimensions and channels, which leads to the generation of a large number of parameters if GRU is used directly. In [33], a ConvLSTM model was proposed, which uses convolution to replace the full connection layer in LSTM. In this way, it captures spatial features by using convolution operations in multi-dimensional data. Furthermore, it avoids the problem of directly using LSTM to generate a large number of parameters. ConvGRU [34] can be implemented by modifying ConvLSTM and converting the LSTM into GRU for calculation. Its calculation method is as follows:

(13) $$z_{t}^{l}=\sigma \left(W_{z}^{l}*x_{t}^{l}+U_{z}^{l}*h_{t-1}^{l}\right)$$

(14) $$r_{t}^{l}=\sigma \left(W_{r}^{l}*x_{t}^{l}+U_{r}^{l}*h_{t-1}^{l}\right)$$

(15) $${\tilde{h}}_{t}^{l}=\tanh\left(W^{l}*x_{t}^{l}+U*\left(r_{t}^{l}⊙h_{t-1}^{l}\right)\right.$$

(16) $$h_{t}^{l}=\left(1-z_{t}^{l}\right)h_{t-1}^{l}+z_{t}^{l}{\tilde{h}}_{t}^{l}$$

where * is the convolution operation, and $W^{l}$, $W_{z}^{l}$, $W_{r}^{l}$, *U*, $U_{r}^{l}$, $U_{z}^{l}$ are all 2D convolution kernels. In order to ensure that the spatial size of the hidden representation remains fixed over time in the model, zero padding is used in the recurrent convolution.

A good time context can effectively guide the network to learn the changes of face skin color caused by pulse changes and suppress the noise caused by motion artifacts and illumination changes. Although 3DCNN has a good ability to learn short-term temporal context, its ability to learn long-term temporal context is relatively weak. In order to improve the network’s ability to learn long-term temporal context and make full use of temporal information, ConvGRU is used at the end of ESA-rPPGNet to further process the temporal features extracted by the feature extraction network.

### 3.5. ESA-rPPGNet

**Figure 10:**
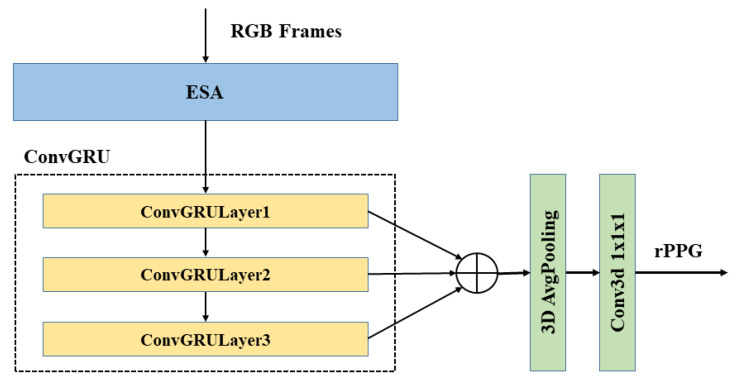
Structure of ESA-rPPGNet. ESA is a spatiotemporal feature extraction network composed of 3DCNN. ConvGRU is a recurrent convolutional neural network proposed in Chapter 3. Here, 3D AvgPooling is used to reduce the spatial dimension of feature map to one. Conv3d is a 3D convolution used to obtain the final rPPG signal.

The overall network structure of ESA-rPPGNet is shown in Figure 10 (Detailed parameter settings can be found in Table 1).

**Figure 11:**
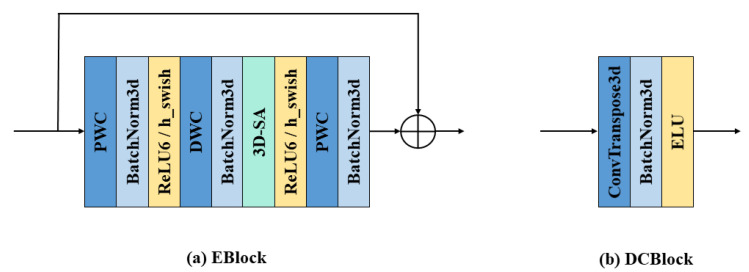
The basic blocks of ESA-rPPGNet. (**a**) is the basic component block of the encoder. (**b**) is the basic component block of the decoder.

In ESA-rPPGNet, ESA is mainly used to accurately extract the spatiotemporal features from the input video to recover the rPPG signal. It is constructed based on the mobilenet v3 structure [35] as a reference. The basic blocks of the network are shown in Figure 11.

The encoder-decoder structure [36] is used in the ESA network. First, the temporal dimension is compressed in the front part of the network, and the DCBlock is used at the end of the network to recover the temporal dimension to the original length. Through this encoder-decoder structure, semantic features with less temporal redundancy can be extracted.

Eblock is the basic component module of the encoder which uses the inverted residual structure proposed in mobilenet v2 [37]. In the inverted residual, the number of channels is first expanded by a PWC. Then, features are extracted through DWC. Finally, the feature maps obtained in the previous step are weighted and fused in the depth direction by PWC, and the number of channels is compressed. The lower the dimension of the tensor, the smaller the multiplication calculation of the convolutional layer, which can effectively improve the overall calculation speed, but may reduce the accuracy of the network. If the filters of the convolutional layer all use low-dimensional tensors to extract features, then there is no way to extract enough information as a whole. By expanding the number of channels first and then compressing the number of channels, the quality of the network and the amount of calculation can reach a balance.

Regarding the nonlinear layer used in the network, it was proved in [38] that a nonlinear layer called swish can significantly improve the accuracy of the network. This nonlinear layer is defined as:

(17) swish(x)=x·σ(x)

where σ(.) is the sigmoid activation function.

Although this nonlinear layer can improve accuracy, the cost of calculating the sigmoid function on a mobile device is high. For this reason, mobilenet v3 proposes two methods to solve this problem:

Firstly, $\frac{ReLU6(x+3)}{6}$ is used to replace the sigmoid function, so that the hardware version of swish becomes

(18) $$h_{-}\mathrm{swish}\left(x\right)=x\frac{ReLU6(x+3)}{6}.$$

Secondly, because the cost of applying swish’s nonlinear layer continues to decrease with the deepening of the network, using swish in the deep layers of the network can bring better results.

According to the above two points, the front part of the ESA network uses ReLU6, and the latter part uses h-swish as the nonlinear layer.

There is a layer of lightweight 3D-SA attention block between DWC and PWC in Eblocks. The 3D-SA module enables the network to focus on important information and fully learn and absorb it, improving the accuracy of the network without bringing too much complexity to the network.

Through the feature extraction network ESA, rich temporal and spatial features are obtained. In order to further strengthen the network’s long-term spatiotemporal feature learning ability, ESA-rPPGNet introduces the ConvGRU module [34]. Three layers of ConvGRU are used in the end of the network; the output of each layer is fused, and then the spatial dimension is pooled through a 3D adaptive average pooling layer. Since ESA-rPPGNet is designed as a fully convolutional structure, the rPPG signal is finally obtained by 3D convolution with a convolution kernel size of 1 × 1 × 1.

### 3.6. Loss Function

After designing our network, we need a suitable loss function to guide the training of ESA-rPPGNet. For the measurement task of HRV, our most important goal is to be able to obtain the rPPG signal with the same trend as the ground truth PPG signal and accurately obtain the peak time locations.

The correlation coefficient is often used to measure the linear correlation between two variables $X\in [x_{1},\ldots ,x_{T}]$ and $Y\in [y_{1},\ldots ,y_{T}]$, and its value is between −1 and 1. Its calculation formula is as follows:

(19) $$\rho_{X,Y}=\frac{\mathrm{con}(X,Y)}{\sigma_{X}\sigma_{Y}}=\frac{E\left[\left(X-\mu_{X}\right)\left(Y-\mu_{Y}\right)\right]}{\sigma_{X}\sigma_{Y}}$$

Equation (19) defines the overall correlation coefficient. The Pearson correlation coefficient can be obtained by estimating the covariance and standard deviation of the sample, and r is commonly used to represent:

(20) $$r=\frac{T\sum_{i=1}^{T}x_{i}y_{i}-\sum_{i=1}^{T}x_{i}\sum_{i=1}^{T}y_{i}}{\sqrt{\left(T\sum_{i=1}^{T}x_{i}^{2}-{\left(\sum_{i=1}^{T}x_{i}\right)}^{2}\right)\left(T\sum_{i=1}^{T}y_{i}^{2}-{\left(\sum_{i=1}^{T}y_{i}\right)}^{2}\right)}}$$

The negative Pearson correlation coefficient is used as the loss function of ESA-rPPGNet to obtain the highest trend similarity and the smallest peak positioning error with the ground truth PPG signal:

(21) Loss=1−r

### 3.7. Signal Post-Processing

Although the quality of the rPPG signal extracted from the video is improved by our method, the noise caused by motion artifacts and illumination changes cannot be completely avoided in the extracted rPPG signal. In order to improve the accuracy of the peak position detected by the peak detection algorithm, we use a band-pass finite impulse response (FIR) filter with a cutoff frequency range of 0.5–3.5 Hz to filter the extracted rPPG signal to obtain a filtered rPPG signal. According to [39], a sampling frequency of 250 Hz was found to be acceptable for HRV analysis. In order to be able to provide sufficient peak accuracy for HRV analysis, the ground truth PPG signal and the filtered rPPG signal we obtained are interpolated with a cubic spline function at a sampling frequency of 256 Hz. When this is done, we can locate the peak position in the signal through the peak detection algorithm. According to the position of the peak, we extract the NNI from the signal, which are the time intervals between consecutive beats.

## 4. Experiments and Result

### 4.1. Datasets

**Figure 12:**
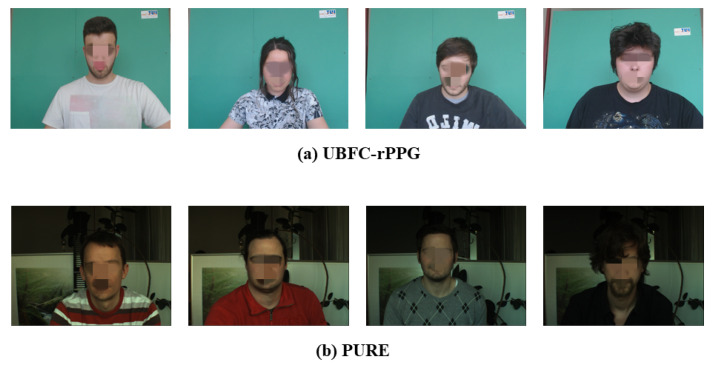
Example images from the two datasets used in this paper.

Two datasets are used in this paper, namely UBFC-rPPG [20] and PURE [18]. The UBFC dataset was created in 2019 and contains 42 videos in total. The dataset uses an uncompressed 8-bit RGB format with a resolution of 640 × 480 to record the subjects’ facial video in the real environment. At the same time, the real-time PPG data of the subjects were recorded through the CMS50E transmission oximeter at 60 Hz. The PURE dataset was created in 2014 and contains a total of 60 videos. The dataset uses an eco274CVGE camera with a resolution of 640 ×480 to record 6 different activities (sitting, talking, rotating and moving head in four ways) of the 10 subjects’ facial videos at 30 fps. Meanwhile, the real-time PPG data of the subjects were recorded through the CMS50E transmission oximeter at 30 Hz. In the UBFC dataset we used, in order to reduce the impact of motion artifacts, the subjects kept as still as possible during recording. To increase task difficulty, subjects in the PURE dataset were recorded with head movements and talk. The examples of the two datasets are shown in Figure 12.

### 4.2. Experimental Setup

The algorithm in this paper is implemented by PyTorch using a Quadro P6000 GPU for training. All CNNs are trained for 30 epochs, adam is used as the optimizer, the initial learning rate is set to 0.001, and it is reduced to 10 epochs with a factor of $10^{-1}$. For all experiments, our batch size is set to 8, the length of each video clip is set to 128 frames, and the step size between each video clip is 8 frames. The frame is resized to 128 × 128. All videos are down-sampled to 30 fps, and real PPG signals are all down-sampled to 30 Hz. We input the video into the network 128 frames at a time, and only after obtaining the rPPG signal extracted from the whole video do we perform HRV analysis.

### 4.3. Evaluation Metrics

Here, several commonly metrics are used for HRV analysis [19,21,22,23,25,40,41]. The metrics used for time domain evaluation of HRV are AVNN, SDNN and RMSSD, their definitions are as follows:

(22) $$\;\mathrm{AVNN}\;=\frac{1}{T}\sum_{i=0}^{T}NN_{i}$$

(23) $$\;\mathrm{SDNN}\;=\sqrt{\frac{1}{T-1}\sum_{i=0}^{T}\left(NN_{i}-AVNN\right)}$$

(24) $$\mathrm{RMSSD}=\sqrt{\frac{1}{T-1}\sum_{i=0}^{T-1}{\left(NN_{i}-NN_{i+1}\right)}^{2}}$$

where AVNN is the average of all NN intervals, SDNN is the standard deviation of all NN intervals, $NN_{i}$ is the *i*-th NN intervals, and T is the number of NN intervals.

For the frequency domain evaluation of HRV, we calculate the three commonly used HRV frequency domain metrics LF, HF and LF/HF in [40] and normalize the unit to n.u. Details about these metrics can be found in [40].

Mean absolute error (MAE) is used to evaluate the performance of HRV’s time domain metrics. Root-mean-square error (RMSE) and Pearson correlation coefficient (R) are used to evaluate the performance of HRV frequency domain metrics. At the same time, in order to evaluate the complexity of the network, this article also evaluates the floating point operations (FLOPs) and parameters of the 3DCNN network.

### 4.4. Experimental Results

This paper tested ESA-rPPGNet on the UBFC-rPPG and PURE datasets, respectively. The test results of time domain and frequency domain metrics on the UBFC-rPPG dataset are shown in Table 2 and Table 3. The test results of time domain metrics and frequency domain metrics on the PURE dataset are shown in Table 4 and Table 5.

**Figure 13:**
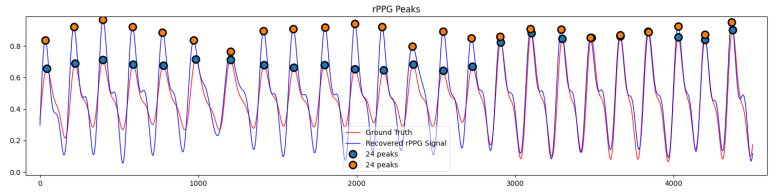
Comparison of peak detection between recovered rPPG signal and ground truth PPG signal.

Due to the precise extraction of temporal and spatial dimensional features of the 3DCNN and 3D-SA attention modules and the ability of ConvGRU to learn long-term spatiotemporal features, the ESA-rPPGNet proposed in this paper can accurately learn the peak time locations of the rPPG signal, as shown in Figure 13. This is particularly important for the measurement of HRV. The estimation of the time-domain and frequency-domain metrics of HRV on the UBFC dataset has achieved better results than other methods, including PhysNet, which also uses 3DCNN.

**Figure 14:**
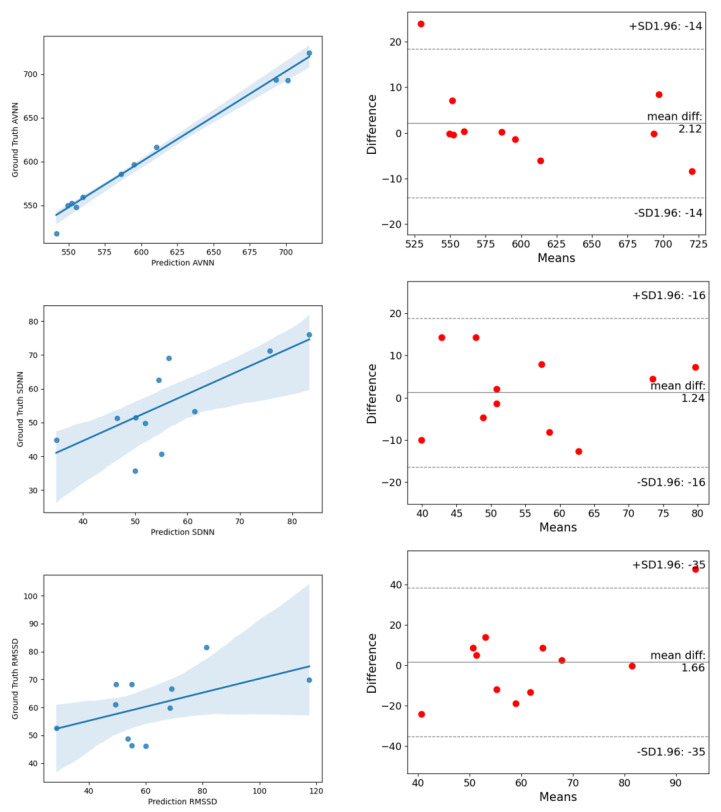
The analysis of the correlation plot (**left**) and the Bland–Altman plot (**right**) of time-domain metrics of HRV on the UBFC dataset. (**top**) is AVNN, (**middle**) is SDNN, (**bottom**) is RMSSD.

**Figure 15:**
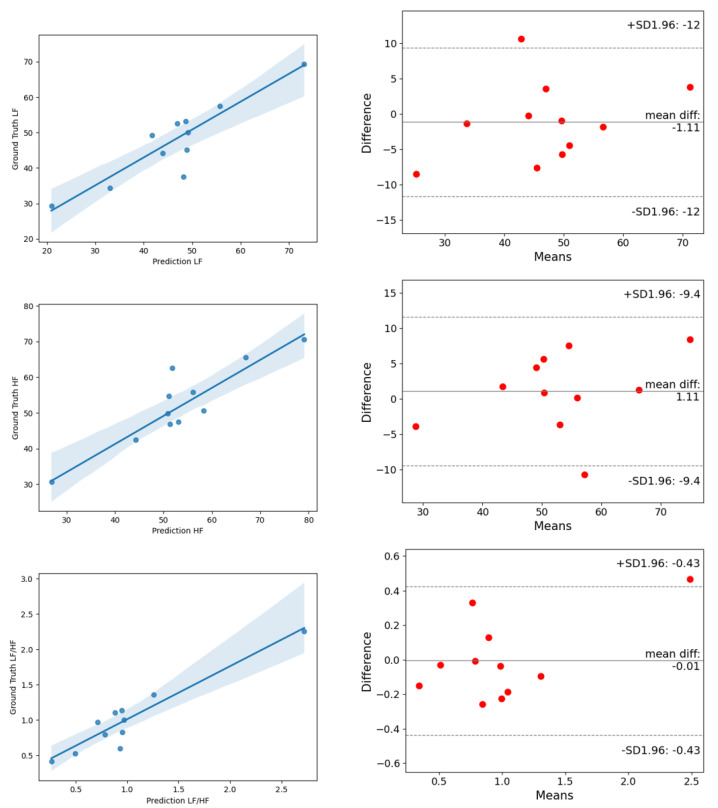
The analysis of the correlation plot (**left**) and the Bland–Altman plot (**right**) of frequency-domain metrics of HRV on the UBFC dataset. (**top**) is LF, (**middle**) is HF, (**bottom**) is LF/HF.

On the UBFC dataset, the estimated MAE of AVNN by ESA-rPPGNet is 5.144, the MAE of SDNN is 13.761 and the MAE of RMSSD is 14.17. These values are lower than existing methods, showing the effectiveness of the method in this paper in the time domain evaluation of HRV. In the frequency domain of HRV, this paper uses the total power of low-frequency, LF (0.04–0.15Hz), the total power of high-frequency, HF (0.15-0.4Hz), and their two ratios LF/HF for evaluation, where LF and HF are divided by the sum of LF and HF to the standard unit (u.n). For LF and HF, between the estimated value and the ground truth, the RMSE is 0.0546, and R is 0.9018. The RMSE of LF/HF is 0.2191, and R is 0.9424. It can be seen that the root-mean-square error of the method in this article is lower than the existing methods, and the Pearson correlation coefficient is higher than the existing methods, which shows that the method proposed in this paper is also very effective for the measurement of HRV in the frequency domain. In order to be able to more clearly reflect the difference between the results predicted by ESA-rPPGNet and the ground truth, Figure 14 and Figure 15 show the analysis of correlation plot and the Bland–Altman plot, respectively.

**Figure 16:**
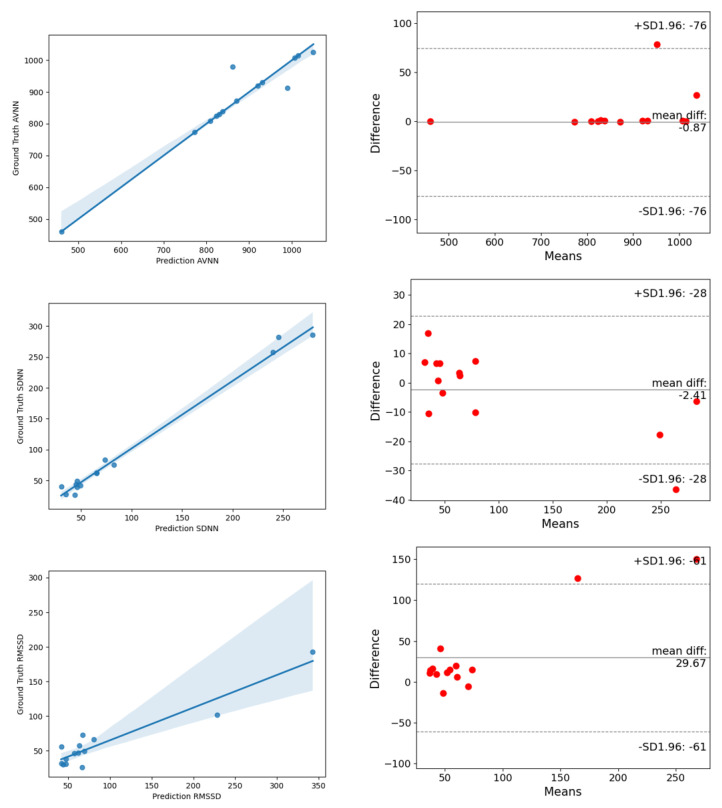
The analysis of the correlation plot (**left**) and the Bland–Altman plot (**right**) of time-domain metrics of HRV on the PURE dataset. (**top**) is AVNN, (**middle**) is SDNN, (**bottom**) is RMSSD.

**Figure 17:**
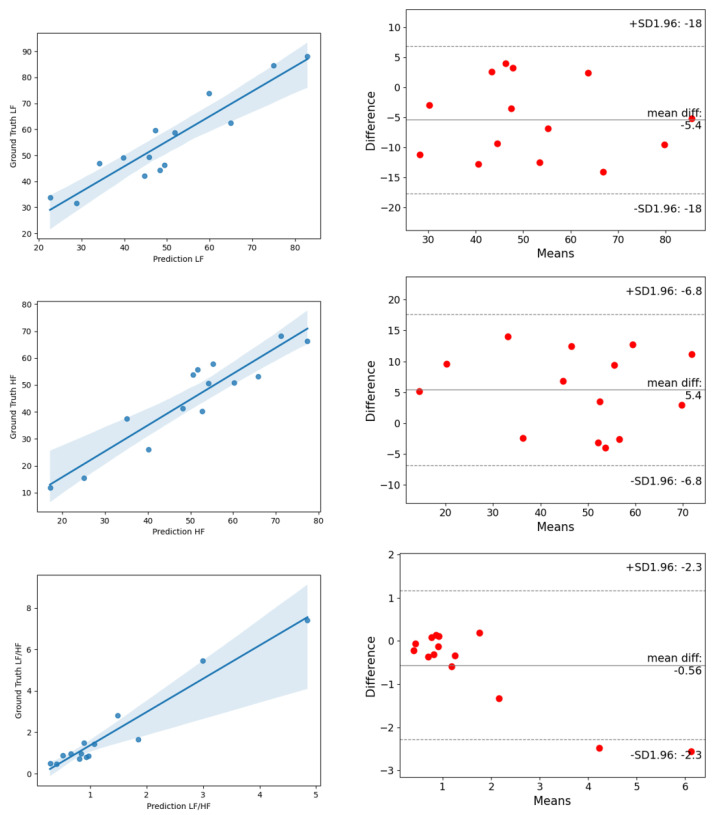
The analysis of the correlation plot (**left**) and the Bland–Altman plot (**right**) of frequency-domain metrics of HRV on the PURE dataset. (**top**) is LF, (**middle**) is HF, (**bottom**) is LF/HF.

In order to further verify the generalization ability of ESA-rPPGNet, this paper also carried out the time-domain and frequency-domain metrics evaluation of HRV on the PURE dataset. The results are shown in Table 4 and Table 5. The analysis of the correlation plot and the Bland-Altman plot for metrics of HRV are shown in Figure 16 and Figure 17. The MAE of AVNN, SDNN are 8.92, 11.75 and 26.12, respectively. For LF and HF, the RMSE is 0.0824, and R is 0.9284. The RMSE of LF/HF is 1.0441, and R is 0.9699. It can be seen that our method has also achieved very good results on the PURE dataset.

Although 3DCNN has natural advantages for the extraction of temporal features, it increases the time complexity of the network. Because the computing power of most devices in real life is limited, the time complexity plays a key role in whether the non-contact physiological measurement can really be applied in life. At present, 3DCNN does not have a more efficient network architecture. Inspired by mobilenet in 2DCNN, this paper uses 3D deep-wise separable convolution and constructs ESA-rPPGNet based on the mobilenet v3 structure. Under the same conditions (input sizes are 1 × 3 × 128 × 128 × 128, which are, respectively, batch size, number of channels, number of video frames, height and width), compared to PhysNet’s 3DCNN structure, our FLOPs are greatly reduced; although the amount of parameters has been improved, they are still in the acceptable range, as shown in Table 6.

## 5. Conclusions and Discussion

Compared with the non-contact measurement of HR, the non-contact measurement of HRV is very sensitive to noise. In this paper, an HRV analysis method is proposed which effectively improves the network temporal context learning ability by using an efficient spatiotemporal attention network ESA-rPPGNet. The network can learn the skin color changes caused by pulse changes and suppress noise caused by motion artifacts and illumination changes. Excellent results have been achieved on the UBFC and PURE datasets. At the same time, ESA-rPPGNet also introduces 3D depth-wise separable convolution, which actively reduces the time complexity of the network and makes up for the shortcomings of the relatively high time complexity of the 3DCNN. This makes it possible to perform non-contact physiological measurement on resource-constrained devices. The method proposed in this paper lays a foundation for the application of non-contact physiological measurement technology in daily life.

Non-contact HRV analysis based on deep learning has only begun to gain attention in recent years. There are still many problems waiting for us to solve. Most of the current methods focus on the measurement of LFnu, HFnu and LF/HF. However, these indicators are far from enough for HRV analysis. More HRV indicators which are better focused on autonomic cardiac control, such as the raw HF power and pNN50, should be considered for validating the proposed method. In addition, many methods cannot be compared fairly now because they mostly use small, private, self-collected datasets, as there is no public benchmark database for evaluation. ECG as the golden metric for HRV analysis should also be included in the dataset. In future work, we will address these issues and explore how to further reduce the noise caused by motion artifacts and illumination changes in non-contact physiological measurements.

## Figures and Tables

**Table 1:** The details of the ESA-rPPGNet network structure. Input is the input dimension of each layer H×W×T×C, Operator is the convolution operation in each layer and the corresponding convolution kernel size, and Exp size is the channel of DWC. Opt size is the final output channel number of the Block, Stride is the step size of the DWC, SA indicates whether to use the 3D-SA module, where 1 indicates yes, 0 indicates no, and NL indicates the nonlinear layer type: 0 means ReLU6, 1 means $h_{-}\mathrm{swish}$.

Input	Operator	Exp Size	Opt Size	Stride	SA	NL
$128^{2}\times 128\times 3$	Conv3d, 3 × 3 × 3	-	16	1 × 2 × 2	-	-
$64^{2}\times 128\times 16$	EBlock, 3 × 3 × 3	16	16	1 × 2 × 2	1	0
$32^{2}\times 128\times 16$	EBlock, 3 × 3 × 3	72	24	1 × 2 × 2	0	0
$16^{2}\times 128\times 24$	EBlock, 3 × 3 × 3	88	24	1 × 1 × 1	0	0
$16^{2}\times 128\times 24$	EBlock, 5 × 5 × 5	96	40	2 × 2 × 2	1	1
$8^{2}\times 64\times 40$	EBlock, 5 × 5 × 5	240	40	1 × 1 × 1	1	1
$8^{2}\times 64\times 40$	EBlock, 5 × 5 × 5	240	40	1 × 1 × 1	1	1
$8^{2}\times 64\times 40$	EBlock, 5 × 5 × 5	120	48	1 × 1 × 1	1	1
$8^{2}\times 64\times 48$	EBlock, 5 × 5 × 5	144	48	1 × 1 × 1	1	1
$8^{2}\times 64\times 48$	EBlock, 5 × 5 × 5	288	96	2 × 2 × 2	1	1
$4^{2}\times 32\times 96$	EBlock, 5 × 5 × 5	576	96	1 × 1 × 1	1	1
$4^{2}\times 32\times 96$	EBlock, 5 × 5 × 5	576	96	1 × 1 × 1	1	1
$4^{2}\times 32\times 96$	Conv3d, 1 × 1 × 1	-	576	1 × 1 × 1	-	-
$4^{2}\times 32\times 576$	DCBlock, 4 × 1 × 1	-	288	2 × 1 × 1	-	-
$4^{2}\times 64\times 288$	DCBlock, 4 × 1 × 1	-	144	2 × 1 × 1	-	-
$4^{2}\times 128\times 144$	ConvGRU	-	64	-	-	-
$4^{2}\times 128\times 64$	GAP	-	64	-	-	-
$1^{2}\times 128\times 64$	Conv3d, 1 × 1 × 1	-	1	1 × 1 × 1	-	-

**Table 2:** The result of time-domain metrics on the UBFC-rPPG dataset.

Method	${\mathrm{AVNN}}_{\mathrm{mae}}\left(\mathrm{ms}\right)$	${\mathrm{SDNN}}_{\mathrm{mae}}\left(\mathrm{ms}\right)$	${\mathrm{RMSSD}}_{\mathrm{mae}}\left(\mathrm{ms}\right)$
CHROM [9]	16.54	40.90	93
SSF [19]	-	25	47
FaceRPPG [21]	-	19	16
PulseGAN [22]	7.52	18.36	-
PhysNet [25]	8.23	15.12	32.58
Ours	5.14	13.76	14.17

**Table 3:** The result of frequency-domain metrics on the UBFC-rPPG dataset.

Method	LF (u.n)		HF (u.n)		LF/HF	
	RMSE	R	RMSE	R	RMSE	R
POS [10]	0.169	0.479	0.169	0.479	0.399	0.518
CHROM [9]	0.240	0.159	0.240	0.159	0.645	0.226
Green [41]	0.186	0.280	0.186	0.280	0.365	0.492
FaceRPPG [21]	0.2	-	0.2	-	1.0	-
CVD [23]	0.065	0.740	0.065	0.740	0.168	0.812
PhysNet [25]	0.0984	0.8024	0.0984	0.8024	0.9734	0.7991
Ours	0.0546	0.9018	0.0546	0.9018	0.2191	0.9424

**Table 4:** The result of time-domain metrics on the PURE dataset.

Method	${\mathrm{AVNN}}_{\mathrm{mae}}\left(\mathrm{ms}\right)$	${\mathrm{SDNN}}_{\mathrm{mae}}\left(\mathrm{ms}\right)$	${\mathrm{RMSSD}}_{\mathrm{mae}}\left(\mathrm{ms}\right)$
CHROM [9]	49.63	89.30	-
FaceRPPG [21]	-	18	15
PulseGAN [22]	28.92	49.39	-
PhysNet [25]	12.34	14.22	34.16
Ours	8.92	11.75	26.12

**Table 5:** The result of frequency-domain metrics on the PURE dataset.

Method	LF (u.n)		HF (u.n)		LF/HF	
	RMSE	R	RMSE	R	RMSE	R
FaceRPPG [21]	0.1	-	0.1	-	1.3	-
PhysNet [25]	0.1115	0.8413	0.1115	0.8413	1.0710	0.8891
Ours	0.0824	0.9284	0.0824	0.9284	1.0441	0.9699

**Table 6:** Comparison of FLOPs and parameters based on 3DCNN method.

Method	FLOPs (G)	Params (M)
PhysNet	115.16	0.77
Ours	4.69	1.62

## References

[1] Dekker J.M., Schouten E.G., Klootwijk P., Pool J., Swenne C.A., Kromhout D. (1997). Heart rate variability from short electrocardiographic recordings predicts mortality from all causes in middle-aged and elderly men: The Zutphen Study. Am. J. Epidemiol..

[2] Kranjec J., Beguš S., Geršak G., Drnovšek J. (2014). Non-contact heart rate and heart rate variability measurements: A review. Biomed. Signal Process. Control.

[3] Hassan M.A., Malik A.S., Fofi D., Saad N., Karasfi B., Ali Y.S., Meriaudeau F. (2017). Heart rate estimation using facial video: A review. Biomed. Signal Process. Control.

[4] Al-Naji A., Gibson K., Lee S.H., Chahl J. (2017). Monitoring of cardiorespiratory signal: Principles of remote measurements and review of methods. IEEE Access.

[5] Balakrishnan G., Durand F., Guttag J. Detecting pulse from head motions in video. Proceedings of the IEEE Conference on Computer Vision and Pattern Recognition.

[6] Li X., Chen J., Zhao G., Pietikainen M. Remote heart rate measurement from face videos under realistic situations. Proceedings of the IEEE Conference on Computer Vision and Pattern Recognition.

[7] Poh M.Z., McDuff D.J., Picard R.W. (2010). Non-contact, automated cardiac pulse measurements using video imaging and blind source separation. Opt. Express.

[8] Lewandowska M., Rumiński J., Kocejko T., Nowak J. (2011). Measuring pulse rate with a webcam—A non-contact method for evaluating cardiac activity. Proceedings of the 2011 Federated Conference on Computer Science and Information Systems (FedCSIS).

[9] De Haan G., Jeanne V. (2013). Robust pulse rate from chrominance-based rPPG. IEEE Trans. Biomed. Eng..

[10] Wang W., den Brinker A.C., Stuijk S., De Haan G. (2016). Algorithmic principles of remote PPG. IEEE Trans. Biomed. Eng..

[11] Gudi A., Bittner M., van Gemert J. (2020). Real-Time Webcam Heart-Rate and Variability Estimation with Clean Ground Truth for Evaluation. Appl. Sci..

[12] Chen W., McDuff D. Deepphys: Video-based physiological measurement using convolutional attention networks. Proceedings of the European Conference on Computer Vision (ECCV).

[13] Špetlík R., Franc V., Matas J. Visual heart rate estimation with convolutional neural network. Proceedings of the British Machine Vision Conference.

[14] Niu X., Han H., Shan S., Chen X. (2018). Synrhythm: Learning a deep heart rate estimator from general to specific. Proceedings of the 2018 24th International Conference on Pattern Recognition (ICPR).

[15] Lee E., Chen E., Lee C.Y. (2020). Meta-rppg: Remote heart rate estimation using a transductive meta-learner. Proceedings of the European Conference on Computer Vision.

[16] Rodríguez A.M., Ramos-Castro J. (2018). Video pulse rate variability analysis in stationary and motion conditions. Biomed. Eng. Online.

[17] Finžgar M., Podržaj P. (2020). Feasibility of assessing ultra-short-term pulse rate variability from video recordings. PeerJ.

[18] Stricker R., Müller S., Gross H.M. (2014). Non-contact video-based pulse rate measurement on a mobile service robot. Proceedings of the 23rd IEEE International Symposium on Robot and Human Interactive Communication.

[19] Li P., Benezeth Y., Nakamura K., Gomez R., Li C., Yang F. (2019). An improvement for video-based heart rate variability measurement. Proceedings of the the 2019 IEEE 4th International Conference on Signal and Image Processing (ICSIP).

[20] Bobbia S., Macwan R., Benezeth Y., Mansouri A., Dubois J. (2019). Unsupervised skin tissue segmentation for remote photoplethysmography. Pattern Recognit. Lett..

[21] Gudi A., Bittner M., Lochmans R., van Gemert J. Efficient real-time camera based estimation of heart rate and its variability. Proceedings of the IEEE/CVF International Conference on Computer Vision Workshops.

[22] Song R., Chen H., Cheng J., Li C., Liu Y., Chen X. (2021). PulseGAN: Learning to generate realistic pulse waveforms in remote photoplethysmography. IEEE J. Biomed. Health Informatics.

[23] Niu X., Yu Z., Han H., Li X., Shan S., Zhao G. (2020). Video-based remote physiological measurement via cross-verified feature disentangling. Proceedings of the European Conference on Computer Vision.

[24] Ji S., Xu W., Yang M., Yu K. (2012). 3D convolutional neural networks for human action recognition. IEEE Trans. Pattern Anal. Mach. Intell..

[25] Yu Z., Li X., Zhao G. (2019). Remote photoplethysmograph signal measurement from facial videos using spatio-temporal networks. arXiv.

[26] Zhang K., Zhang Z., Li Z., Qiao Y. (2016). Joint face detection and alignment using multitask cascaded convolutional networks. IEEE Signal Process. Lett..

[27] Hu J., Shen L., Sun G. Squeeze-and-excitation networks. Proceedings of the IEEE Conference on Computer Vision and Pattern Recognition.

[28] Woo S., Park J., Lee J.Y., Kweon I.S. Cbam: Convolutional block attention module. Proceedings of the European Conference on Computer Vision (ECCV).

[29] Zhang Q.L., Yang Y.B. (2021). Sa-net: Shuffle attention for deep convolutional neural networks. Proceedings of the ICASSP 2021–2021 IEEE International Conference on Acoustics, Speech and Signal Processing (ICASSP).

[30] Ma N., Zhang X., Zheng H.T., Sun J. Shufflenet v2: Practical guidelines for efficient cnn architecture design. Proceedings of the European Conference on Computer Vision (ECCV).

[31] Wu Y., He K. Group normalization. Proceedings of the European Conference on Computer Vision (ECCV).

[32] Chung J., Gulcehre C., Cho K., Bengio Y. (2014). Empirical evaluation of gated recurrent neural networks on sequence modeling. arXiv.

[33] Xingjian S., Chen Z., Wang H., Yeung D.Y., Wong W.K., Woo W.c. (2015). Convolutional LSTM network: A machine learning approach for precipitation nowcasting. Advances in Neural Information Processing Systems.

[34] Ballas N., Yao L., Pal C., Courville A. (2015). Delving deeper into convolutional networks for learning video representations. arXiv.

[35] Howard A., Sandler M., Chu G., Chen L.C., Chen B., Tan M., Wang W., Zhu Y., Pang R., Vasudevan V. Searching for mobilenetv3. Proceedings of the IEEE/CVF International Conference on Computer Vision.

[36] Lea C., Flynn M.D., Vidal R., Reiter A., Hager G.D. Temporal convolutional networks for action segmentation and detection. Proceedings of the IEEE Conference on Computer Vision and Pattern Recognition.

[37] Howard A., Zhmoginov A., Chen L.C., Sandler M., Zhu M. Inverted residuals and linear bottlenecks: Mobile networks for classification, detection and segmentation. Proceedings of the IEEE Conference on Computer Vision and Pattern Recognition.

[38] Ramachandran P., Zoph B., Le Q.V. (2017). Searching for activation functions. arXiv.

[39] Kwon O., Jeong J., Kim H.B., Kwon I.H., Park S.Y., Kim J.E., Choi Y. (2018). Electrocardiogram sampling frequency range acceptable for heart rate variability analysis. Healthc. Inform. Res..

[40] Poh M.Z., McDuff D.J., Picard R.W. (2010). Advancements in noncontact, multiparameter physiological measurements using a webcam. IEEE Trans. Biomed. Eng..

[41] Verkruysse W., Svaasand L.O., Nelson J.S. (2008). Remote plethysmographic imaging using ambient light. Opt. Express.

